# Biotechnological Preparation of Gelatines from Chicken Feet

**DOI:** 10.3390/polym11061060

**Published:** 2019-06-18

**Authors:** Pavel Mokrejš, Petr Mrázek, Robert Gál, Jana Pavlačková

**Affiliations:** 1 Department of Polymer Engineering, Faculty of Technology, Tomas Bata University in Zlín, Vavrečkova 275, 760 01 Zlín, Czech Republic; p_mrazek@utb.cz; 2 Department of Food Technology, Faculty of Technology, Tomas Bata University in Zlín, Vavrečkova 275, 760 01 Zlín, Czech Republic; gal@utb.cz; 3 Department of Lipids, Detergents and Cosmetics Technology, Faculty of Technology, Tomas Bata University in Zlín, Vavrečkova 275, 760 01 Zlín, Czech Republic; pavlackova@utb.cz

**Keywords:** biotechnology, by-products, chicken feet, extraction, food applications, gelatine, pharmaceutical applications, polymer biomaterials

## Abstract

In the European Union (EU), about five tons of poultry by-product tissues are produced every year. Due to their high collagen content, they represent a significant raw material source for gelatine production. The aim of the paper was the biotechnological preparation of gelatine from chicken feet. The influence of selected process factors on the gelatine yield, gel strength, viscosity, and ash of gelatine was observed; a two-level factor design of experiments with three variable process factors (enzyme addition, enzyme treatment time, and gelatine extraction time) was applied. After grinding and separating soluble proteins and fat, the purified raw material was treated in water at pH 7.5 with the addition of endoprotease at 23 °C and after thorough washing with water at 80 °C, gelatine was extracted. By the suitable choice of process conditions, gelatine with high gel strength (220–320 bloom), low ash content (<2.0%) and viscosity of 3.5–7.3 mPa·s can be prepared. The extraction efficiency was 18–38%. The presented technology is innovative mainly by the enzymatic processing of the source raw material, which is economically, technologically, and environmentally beneficial for manufacturers. Chicken gelatines are a suitable alternative to gelatines made from mammals or fish, and can be used in many food, pharmaceutical, and biomedical applications.

## 1. Introduction

Gelatine is a significant, water-soluble protein that is obtained from collagenous raw materials by partial hydrolysis. The primary raw materials for gelatine production are pork and bovine skins/hides and bones [1]. In recent years, alternative sources of collagen, especially fish and by-products of the meat processing and poultry industry, have become more important for gelatine producers [2]. The reason for this is the growing global demand for gelatine, which is estimated to be around 451,000 tons for 2018: about a ¼ increase over six years [3]. Another impetus for the search for alternative sources of collagen is the growing demand for non-mammalian gelatines, especially from consumers from Islamic, Jewish, and Hindu countries. Also, the economic and ecological reasons for the consideration of by-products are forcing producers of such waste to seek ways of further re-utilization. The new application possibilities of gelatine made from alternative sources of collagen are also opening up.

Worldwide annual food waste production is estimated at up to 100 million tons. In the European Union (EU), the meat processing industry produces 16.5 million tons of waste, and 5.2 million tons of waste is generated in the fish production industry [4]. The United States (US) poultry industry produced 28.4 million tons of live weight of poultry in 2016; the largest proportion (24.9 million tons) was chicken [5]. Poultry meat accounts for 5.5% of total agricultural production and 12.7% of EU meat production. In 2014, 13 million tons of poultry meat was produced in the EU, 78% of which was chicken [6]. According to the FAO (Food and Agricultural Organization), the total consumption of poultry meat is growing by 3.6% per year. Even though in some countries animal by-products (e.g., kidneys, hearts, livers, lungs, stomachs, and tongues) are used for culinary purposes, approximately 20–30% of the live weight of poultry represents unused by-products. In some countries, for example, blood is used as an ingredient in food production and for the production of feeding meal. Feathers and other by-products are used for the production of feeding hydrolysates; part of the protein waste can also be composted or used for fuel production, and waste fats are used for soaps, biofuels, and lubricants [7,8,9,10].

Raw materials for the production of commercial gelatines are processed in low and high pH environments [1]. In the case of type A gelatine, the raw material is treated for 18–30 h in an acidic environment at pH 1.5–3.0; this procedure is suitable for pigskins. For type B gelatine, the raw material is treated in alkaline medium at pH 12.0 for several weeks to months; this procedure is mainly used for cowhides.

By-products from freshwater and saltwater fish rich in collagen (skins and bones) have long been known as the suitable raw materials for gelatines and collagen hydrolysates production. In the preparation of fish gelatines, the procedure is analogous to that of the production of gelatines from mammals. Most often, the raw material is treated for 12–48 h in an acidic environment at 5–10 °C, optionally at room temperature; weak solutions of acids (0.05–0.5 mol·L^−1^) are used, especially acetic acid, citric acid, phosphoric acid, formic acid, hydrochloric acid, propionic acid, and optionally lactic acid. Depending on the type of the raw material (different amounts of intermolecular and intramolecular crosslinking of the collagen matrix) and the extraction conditions (water, usually at 45–55 °C for 3–12 h), gelatine (type A) is prepared with a yield of 5.5–66.0% [11,12,13]. Less common is the processing of the raw material in an alkaline environment, most often using Ca(OH)_2_ (type B gelatine), as well as the use of protease [14,15,16]. Somewhat different is the industrial production of gelatines, soluble collagen, and hydrolysates from poultry tissues. On the global market, chicken products represent only a fraction of gelatines made from beef, pork, and fish. After 24 h of processing of fowl feet with weak acid solutions (0.5 M acetic acid, citric acid, hydrochloric acid, or lactic acid) and enzyme (pepsin) at 4 °C, soluble collagen is prepared with relatively low yields, 5.6–8.4% [17]. Using stronger solutions of the same acids (5.0%, v/v), acid-soluble collagen can be prepared under similar processing conditions (12–36 h incubation at 4–7 °C) with a yield of 7.9–31.2%, even without the use of pepsin [18]. Also, the preparation of gelatines, especially from chicken feet, skins, and tendons, is described. After the removal of soluble non-collagenous proteins and pigments, the feet are processed in an acidic environment using slightly stronger solutions (1.5–4.5%, v/v) of acetic acid, citric acid, or lactic acid at room temperature for 16–18 h. At moderate extraction temperatures (50–55 °C) and extraction times ranging from tens of minutes to hours, gelatine with a gel strength of 120–300 Bloom is prepared, which is comparable to commercial pork and fish gelatines. The disadvantage is the relatively low yields of the prepared gelatines, 6.0–14.5%, based on the dry weight of the source material [19,20]. After combined sulphuric acid and acetic acid treatment and 12-h extraction at 45 °C, 16% of high gel-strength gelatine (355 Bloom) can be obtained from chicken skins, which is more than in conventional commercial bovine gelatine [21]. The literature also describes the processing of other poultry by-products into gelatines. These are mainly chicken or turkey heads or duck feet. The processing is carried out according to an analogous procedure to that of chicken feet or skins. The alkaline method of poultry tissue processing is not widespread, but has been tried, for example, on chicken and turkey heads [22]. 

The processing of some by-products from poultry slaughter into gelatines have been satisfactorily described in the literature. However, all the techniques are based on the acidic or alkaline processing of the raw material; the enzymatic processing is only marginally mentioned. The use of enzymes brings with it many advantages, such as mild reaction conditions given by temperature and pH as well as low doses of enzymes used. The main goal of our paper is to propose a biotechnological method of preparing gelatines from chicken feet, through first treating the source material with a suitable proteolytic enzyme and then extracting the gelatine with hot water. This is followed by observing the influence of selected process factors on gelatine yield (percentage conversion of the source material to gelatines) and the quality of prepared gelatines (gel strength, viscosity, and ash content in gelatine). The specific hypothesis being tested is that chicken feet, after treatment with endoprotease and extraction with hot water, can be processed into gelatines whose properties are comparable with gelatines produced from pork and beef tissues.

## 2. Materials and Methods

**Scheme 1:**
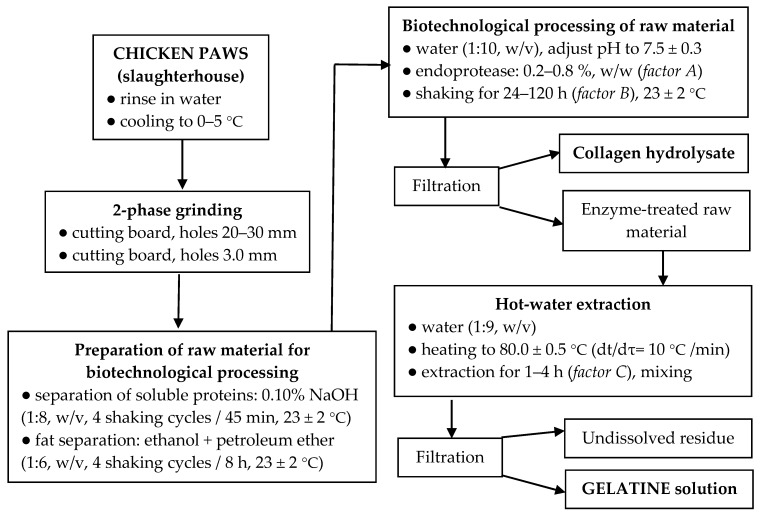
A flow chart of preparation of gelatins from chicken feet.

A flow chart of biotechnological processing of chicken feet into gelatines is shown in Scheme 1.

### 2.1. Materials

Chicken feet were obtained in the Raciola poultry farm (Ltd., Uherský Brod, Czech Republic) and processed under strict hygiene conditions, see Section 2.3 and Section 2.4. Raw chicken feet composition: 35.0 ± 3.0% dry matter content; dry matter: 48.3 ± 0.4% protein content (82.8 ± 0.7% collagen content), 34.8 ± 0.8% fat content, 16.1 ± 0.2% ash content. The polarzyme 6.0 T-granulated endoprotease that hydrolyzes internal peptide bonds were manufactured by the fermentation of a microorganism that is not present in the final product (Novozymes, Copenhagen, Denmark), petrolether, ethanol, NaOH, 36% HCl. The procedure also used a SPAR Mixer SP-100AD-B industrial meat cutting machine with a four-arm knife (Panther T & H Industry, Taichung, Taiwan), Nabertherm muffle furnace (Lilienthal, Germany), WTW pH 526 pH meter (WTW, Oberbayern, Germany), Kavalier LT3 shaker (Sázava, Czech Republic), Kern 770 analytical and precision balances (Balingen, Germany), Kern 440-47 laboratory scales (Kern, Balingen, Germany), MEMMERT ULP 400 dryer (Schwabach, Germany), WTB Binder E-28-TB1 dryer (Tuttlingen, Germany), IKA LABORTECHNIK RCT BASIC magnetic mixer with heating plate (Staufen, Germany), stainless steel sieve with 1.0-mm size, Stevens-LFRA Texture Analyser (Brookfield, UK).

### 2.2. Strategy

A two-stage biotechnological procedure was designed for the conversion of collagen to gelatines, which due to its variability of the process parameters would allow preparing the gelatines of the desired properties with the optimum utilization of the source material. In industrial practice, factor experiments are widely used to design experiments consisting of multiple technological variables, allowing maximum information to be obtained and optimal process conditions to be designed [23].

To study the influence of selected process factors on gelatine yield and the quality of prepared gelatines, 2^3^ factor schemes with one central experiment and one repetition were used; then, in the optimization part of the process, 2^2^ factor schemes with one central experiment and one repetition were used. The process factors studied were: factor A—enzyme addition: a minimum value of 0.2% (based on the protein dry matter, w/w), a mean value of 0.5% (w/w), a maximum value of 0.8% (w/w); factor B—enzyme treatment time: minimum 24 h, mean 72 h, maximum value 120 h; factor C—gelatine extraction time: minimum 1 h, mean 2.5 h, maximum value 4 h. The variables evaluated were gelatine yield (percentage conversion of the starting protein substrate to gelatine), the strength of the gelatine gels, the gelatine viscosity, and the ash content of the gelatines. On the basis of statistical evaluation of the influence of process parameters on the evaluated quantities, the process optimization was carried out. The process was based on monitoring the influence of the two most important process parameters on the evaluated variables and the design of optimal processing conditions for the conversion of collagen protein of chicken feet to high-quality gelatines.

### 2.3. Grinding and Preparation of Raw Material for Biotechnological Processing

When preparing the starting material for further biotechnological processing, it is necessary to set conditions to avoid the denaturation of the collagen protein, convert collagen to gelatine in biotechnological processing with optimum efficiency, and produce quality gelatines. Immediately after separating the feet, the raw material was rinsed in water and cooled to 0–5 °C to prevent negative microbial growth (the chilled raw material can be stored for a maximum of 36 h after slaughter).

Prior to two-phase grinding (on a meat cutter), the feedstock was slightly pre-frozen at −2 to −5 °C in the core of the feedstock. In the first milling process, kidney-shaped cutting plates with a hole size of 20.0–30.0 mm were used, the second was a hole size of 3.0 mm; during grinding, the temperature of the raw material increased to a maximum of 3 °C. If the ground and homogenized raw material is not processed within a maximum of 24 h, it must be wrapped in vacuum containers with a minimum wall thickness of 80 µm and deep-frozen at −36 ± 2 °C; the frozen raw material can be stored at −20 ± 2 °C for up to 24 months. Furthermore, the homogenized raw material needs to be purified from the accompanying non-collagenous components, pigments, and fat. The removal of albumins, globulins, and pigments was based on the earlier procedure [24] with slight modifications. The raw material was mixed with 0.10% NaOH solution in a ratio of 1:8 (w/v) and shaken for 45 min at room temperature; then, the solution was filtered through a 1.0-mm stainless steel sieve, and the raw material was washed with running water; the whole procedure was repeated four times. Then, the raw material was dried in an air circulating oven at 35.0 ± 0.5 °C (36 h).

The defatting of poultry tissues is described in the literature by several methods, such as the Soxhlet extraction process [21], using NaHCO_3_ [24] or by mechanical separation after fat leaching in alkaline medium [25]. Defatting of the raw material was carried out according to the procedure previously described [26]. The dried raw material was mixed with a mixture of ethanol and petroleum ether (1:1, v/v) solvent in a ratio of 1:6 (w/v) and shaken for 32 h at room temperature; after 8 h, the solvent mixture was replaced with a new one (filtration was carried out through a stainless sieve with a size of 1.0 mm). The defatted raw material was spread on a metal sheet, and the solvent residues were evaporated in a fume hood at room temperature.

### 2.4. Biotechnological Processing of Raw Material and Gelatine Extraction

As there is a lack of biotechnological processing of the raw material prior to gelatine extraction in the available literature, such a procedure has been designed and tested in our workplace. We proceeded from the results of our previous research, which focused on the preparation of protein hydrolysates from chicken feet [27]. The defatted raw material was mixed with distilled water in a ratio of 1:10 (w/v); after 30 min, the pH of the mixture was adjusted to 7.5 ± 0.3 (optimal efficiency of the proteolytic enzyme used) by the addition of 10% HCl. After the addition of Polarzyme 6.0 T in an amount according to factor A (enzyme load based on the dry weight of the defatted raw material), the mixture was shaken at room temperature for a time according to factor B. After filtration (through a 1.0-mm stainless steel sieve), the protein hydrolysate solution was first brought to boil and was boiled for 10 min (inactivation of proteolytic enzyme). Then, it was dried (after pouring onto a stainless steel plate) in a thin film in an air-circulating oven at 45.0 ± 1.0 °C (48 h); afterwards, the dried film was scraped off and weighed. The enzyme treated raw material was thoroughly washed on the sieve under running water and then mixed in a beaker with distilled water in a ratio of 1:9 (w/v). The mixture was placed on a hot plate, stirred gently (magnetic stirrer), heated to 80.0 ± 0.5 °C (d*t*/d*τ* = 10 °C/min), and after reaching this temperature, gelatine was extracted according to the factor C, time. After completion of the extraction, the gelatine solution was separated by filtration through a 1.0-mm stainless steel sieve equipped with three layers of polyamide fabric (300-µm pore size). The gelatine solution was brought to the boil and was boiled for 5 min; then, it was poured onto a thin-plate thin film and dried in an air-circulating oven at 45.0 ± 1.0 °C (48 h); afterwards, the dried film was scraped off and weighed. The undissolved residue of the raw material after gelatine extraction was dried in an air circulating oven at 103.0 ± 1.0 °C (for 16 h) and then weighed.

### 2.5. Analytical Methods 

Dry matter, ash, fat, and protein were determined by conventional food methods [28,29,30]. The dry matter was determined by the indirect method of drying the sample for 18 h at 103.0 ± 2.0 °C; the ash was determined gravimetrically after burning and annealing the sample; fat was determined by Soxhlet extraction; nitrogen was determined by the Kjeldahl method, and the protein content was calculated from the determined nitrogen content by multiplying by a factor of 6.25. Collagen content was calculated from the hydroxyproline content (determined colorimetrically after sample hydrolysis in 6 mol·L^−1^ HCl) by multiplying by a factor of eight [31,32]. Gel strength, gelatine viscosity, and pH were determined according to the Official Procedure of the Gelatine Manufacturers Institute of America [33]. The pH of a 1.5% gelatine solution was determined by potentiometry at a temperature of 35 ± 0.5 °C using a pH meter. The gelatine gel strength was determined from a gel formed from a 6.67% solution prepared according to prescribed conditions by the measuring of force (weight) required to depress a prescribed area of the surface of the sample to a distance of 4 mm. The dynamic viscosity of a 6.67% gelatine solution was determined at 60 °C by measuring the flow time of 100 mL of the solution through a standard pipette; the viscosity was calculated from Equation (1). The yield of the hydrolysate was calculated from the weight of the hydrolysate prepared after the biotechnological treatment of the raw material, the yield of gelatine from the weight of the gelatine (both yields based on the weight of the defatted raw material); further, the total yield was calculated; see Equations (2, 3, 4). The mass balance error is expressed by the percentage difference of the dry matter mass balance between the input (defatted raw material) and the outputs (hydrolysate, gelatine, and undissolved residue); see Equation (5).

(1) $$\eta =\left(A\tau -\frac{B}{\tau }\right)\;d$$

(2) $$\mathrm{HY}=\frac{m_{1}}{m_{0}}\;100$$

(3) $$\mathrm{GY}=\frac{m_{2}}{m_{0}}\;100\;(3)$$

(4) $$Y_{\sum }=\mathrm{HY}+\mathrm{GY}$$

(5) $$\mathrm{MBE}=\frac{\left\lfloor \left(m_{1}+m_{2}+m_{3}\right)-m_{0}\right\rfloor }{m_{0}}100$$

where *η* is gelatine viscosity (mPa·s), *A* and *B* are pipette constants, *τ* is efflux time (s), *d* is solution density (for a 6.67% gelatine solution at 60 °C d = 1.003), *HY* is the hydrolysate yield (%), *m_0_* is the weight of the defatted raw material (g), *m_1_* is the weight of the hydrolysate, *GY* is the gelatine yield (%), *m_2_* is the weight of gelatine (g), *m_3_* is the weight of the undissolved residue (g), $Y_{\sum }$ is the total yield (%), and *MBE* is a mass balance error (%).

### 2.6. Statistical Analysis 

A two-level factorial design of experiments and evaluation of the results were carried out with Minitab^®^ 17.2.1 software (Fujitsu Ltd., Tokyo, Japan). Statistical analyses (arithmetic means and standard deviations) were accompanied using Excel 2010 (Microsoft, Inc., Seattle, WA, USA) at the significance level of 5% (*P* < 0.05).

## 3. Results and Discussion

### 3.1. Study of the Influence of Process Factors on the Gelatine Yield and Quality of Prepared Products

A schedule of the experiments and summary results of the processing of chicken feet proteins into gelatine and hydrolysates by two-level factor schemes with three factors of concern are given in Table 1. The pH of the prepared gelatines ranged from 6.0 to 6.4, which corresponds to standards for food and pharmaceutical gelatines where pH 4.0–7.5 is prescribed. The pH of commercially produced porcine gelatines ranges from 5.5 to 6.5, with beef gelatines usually between 5.5–7.0, as well as fish gelatines. All the prepared gelatines were characterized by very low ash content (0.61–1.66%), and thus meet the stringent parameters for food and pharmaceutical gelatines (Food Chemical Codex 10; United States Pharmacopoeia 35 NF 30; European Pharmacopoeia; Japanese Pharmacopoeia 15).

The statistical significance of the studied process factors in the observed limits was evaluated using the standard Fisher’s significance test and the P-values for a 95% confidence level. For the factor schemes used by us, the critical value is *F* = 10.13, so the higher the *F*-value is above the critical value, the greater the influence of the process factor. Similarly, results for *P*-values were evaluated; factors with a value lower than α = 0.05 have an effect on the evaluated variables with 95% probability, and the lower the *P*-value, the greater the influence of the process factor [34]. Table 2 shows the results of analysis of variance for gelatine yield, gelatine gel strength, and gelatine viscosity.

#### 3.1.1. Gelatine Yield

**Figure 1:**
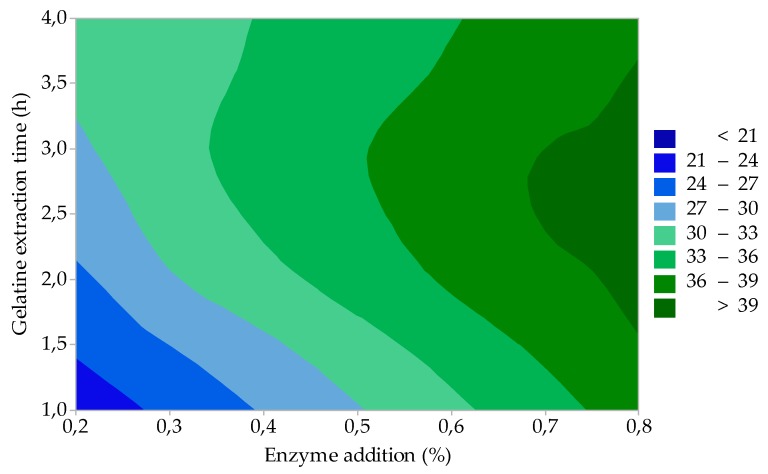
Effect of enzyme addition and gelatine extraction time on gelatine yield (study of the influence of process factors).

The results of the statistical evaluation showed that only factor A (enzyme addition) is statistically significant for the yield of gelatine (GY). The effect of the two most important process factors (enzyme addition and gelatine extraction time) on GY is represented by the contour graph in Figure 1. It is evident that with increasing enzyme addition and at the same time extending the extraction time, GY increases. This trend is particularly evident at lower enzyme additions (up to about 0.4%) and at shorter extraction times (up to about 2.5 h). There is no significant GY growth with any addition of enzymes above 0.5% or extraction time more than 2.5 h. The minimum GY (≈ 21%) is reached under the lower limit of the factors of interest, i.e., 0.2% enzyme addition and 1.0-h extraction time. The maximum GY (≈ 38%) then corresponds to approximately 0.7% enzyme addition and 2.5 h; further increasing the extraction time will no longer affect the increase of GY.

GY values are comparable or better compared to the available results of processing the proteinic poultry tissues into gelatines and hydrolysates. Using an acid extraction procedure and ultrasonic extraction, very low yields of gelatines (4% and 17%) from chicken feet are reported [35]. Almeida, Calarge, and Santana processed chicken feet by pressure extraction in water (120 °C for 20 min) and obtained a 36% yield of low-strength gelatine [36]. Du et al., by processing the chicken and turkey heads in an acidic environment and extracting in two stages (temperatures 50 and 60 °C), prepared high-quality gelatines with yields of 21% and 31% (chicken heads) and 25% and 38% (turkey heads) [24]. Sarbon, Badii, and Howell used the combined alkaline-enzyme processing of raw material to produce high-quality chicken skin gelatine, with a gelatine yield of 16% [21].

#### 3.1.2. Gelatine Gel Strength

**Figure 2:**
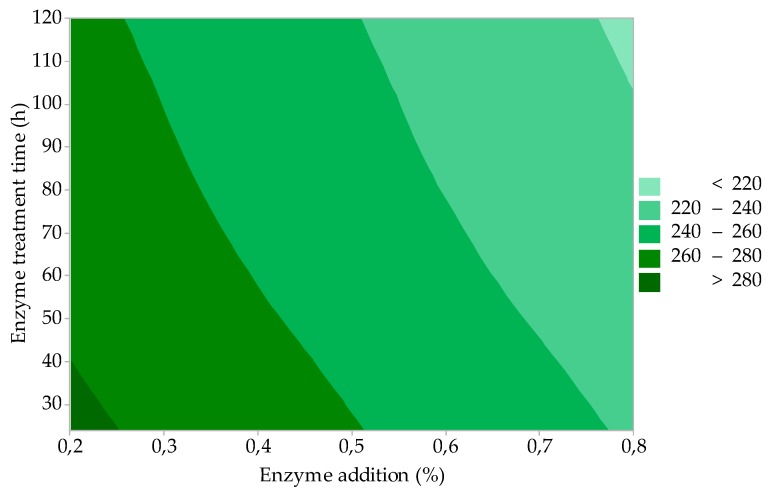
Effect of enzyme addition and enzyme treatment time on gelatine gel strength (study of the influence of process factors).

The results of the statistical evaluation showed that all three studied factors are statistically significant in the strength of gelatine gel (*F*); the influence of factor A (enzyme addition) and factor B (enzyme treatment time) on F is represented by the contour graph in Figure 2. It can be seen from the figure that high-quality gelatines (*F* = 220–280 Bloom) can be prepared within the limits of the process factors studied (0.2–0.8% enzyme addition and raw material enzyme processing for 30 to 120 h). Under the lower limit of the observed factors (0.2% enzyme addition and 30-h enzyme treatment of the raw material, gelatine with *F* > 280 Bloom can be prepared with a 21% yield of gelatine (see Figure 1). With an almost double yield of gelatine (GY = 38%, see Figure 1) under the upper limits of the observed factors (0.8% enzyme addition and 120-h enzyme treatment of the raw material), it is evident that there is no significant decrease in F: gelatine still has a high gel strength (F ≈ 220 Bloom). The standard specifications for food gelatines prescribe *F* = 150–280 Bloom, depending on the method of application. For the production of hard gelatine capsules (HGC), *F* = 200–280 Bloom is prescribed; gelatines with a gel strength of 130–200 Bloom are sufficient for soft gelatine capsules (SGC).

The gel strength of gelatines prepared under various process conditions is the same or higher than that of commercial high-strength gelatines (*F* > 200 Bloom) made from bovine and pork hides/skins and bones and fish. The gelatines prepared by us have a comparable or lower F compared to gelatines prepared from poultry by-products by other authors. Gelatine prepared from chicken feet by acid treatment of the starting material had a gel strength of 295 Bloom [20]. Du et al. reported *F* = 200–248 Bloom in gelatines prepared from chicken heads; however, higher F values (333–368 Bloom) were achieved in gelatines prepared from turkey heads [24]. The results are similar in high-quality gelatine (*F* = 355 Bloom) from chicken skins [21].

#### 3.1.3. Gelatine Viscosity

**Figure 3:**
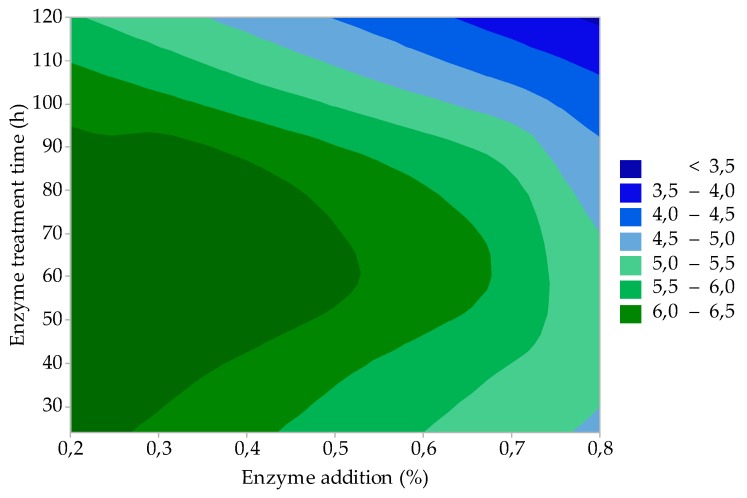
Effect of enzyme addition and enzyme treatment time on the gelatine viscosity (study of the influence of process factors).

The results of statistical evaluation showed that factor A (enzyme addition) and factor B (enzyme treatment time) are statistically significant for gelatine viscosity (η); the influence of these process factors on η is represented by the contour graph in Figure 3. It is evident from the figure that η is the highest (>6.5 mPa·s), and at the same time, its value is not influenced by the change of these technological factors at low enzyme addition (0.2–0.3%) and at short enzymatic treatment times (within 90 h). It is also evident from the graph that with increasing enzyme addition (>0.4%) and at short enzyme treatment times (up to ≈ 90 h), η gradually decreases to 5.0 mPa·s. Long enzyme treatment times (110–120 h) in combination with the highest enzyme additions (0.7–0.8%) mean an even more significant decrease of η (3.5–4.0 mPa·s). Gelatines with η 2.0–7.5 mPa·s cover a wide range of applications in the food industry. For the production of HGC, gelatines with a higher viscosity (4.5–6.5 mPa·s) are required. For SGC, it is 2.5–4.5 mPa·s, and for tablets, the viscosity of 1.7–3.5 mPa·s is sufficient. It can be seen from the viscosity measurement results that gelatines prepared under different process conditions meet the gelatine specifications for the production of HGC and SGC.

### 3.2. Process Optimization

From the study of the influence of process parameters on the gelatine yields and the quality of the prepared gelatines, the following conclusions can be drawn: *a)* with increasing enzyme addition (factor A) and with increasing gelatine extraction time (factor C), the gelatine yield increases; *b)* with increasing enzyme addition (factor A) and with increasing enzyme treatment time (factor B), the strength of the gelatine gels decreases; *c)* the highest gelatine gel strength (≈ 295 Bloom) was recorded under the conditions of the lower process factors observed (0.2% enzyme addition, 24-h enzyme treatment time and 1-h gelatine extraction time); *d)* it can be assumed that the strength of the gelatine gels will grow at lower enzyme additions, shorter enzyme treatment times, and shorter extraction times. 

When optimizing the process conditions for the preparation of gelatines from chicken feet, the goal was to prepare high-quality gelatines (with a gel strength of at least 300 Bloom) even at the cost of a lower gelatine yield. It was decided to enzymatically process the raw material for a constant time (20 h) and monitor the effect of enzyme addition (0.1–0.4%) and gelatine extraction time (15–45 min) on the quality of the prepared gelatines (gel strength, viscosity, ash content) and gelatine yield. A schedule of experiments (factor schemes 2^2^) and summary results of the optimization part of processing chicken feet proteins to gelatines are given in Table 3.

**Figure 4:**
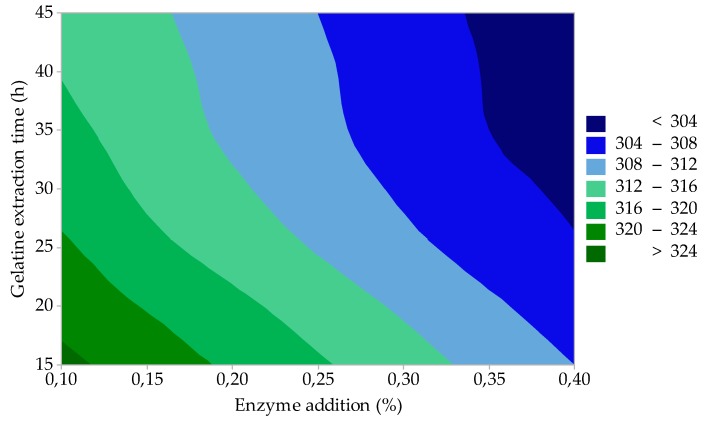
Effect of enzyme addition and gelatine extraction time on gelatine gel strength (process optimization).

**Figure 5:**
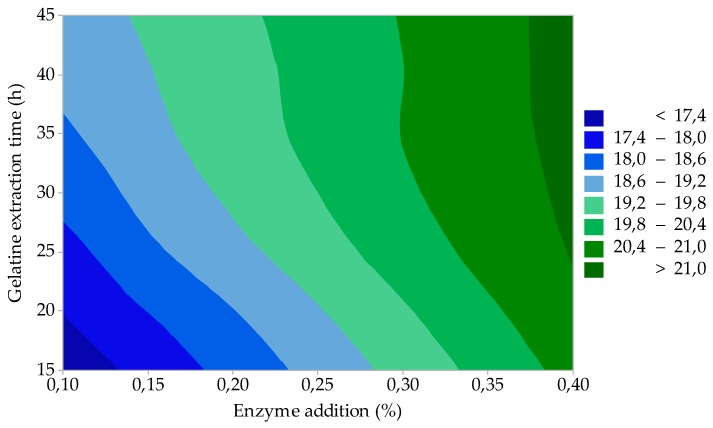
Effect of enzyme addition and gelatine extraction time on gelatine yield (process optimization).

As shown in Figure 4, gelatines with a very high gel strength can be prepared by the appropriate choice of enzyme addition (factor A) and gelatine extraction time (factor C). In the case of low enzyme addition (up to 0.15%) and short extraction times (up to 20 min), the highest quality gelatine (F = 320–325 Bloom) is prepared; the gelatine yield is approximately 17.5–18.0% (see Figure 5). By increasing the enzyme addition to the upper test limit (0.4%) and extending the extraction time (up to 45 min), the gelatine yield increases to about 21.0% (Figure 5), but gelatine still has a very high gel strength (F ≈ 305 Bloom); see Figure 4.

The pH of the prepared gelatines ranged between 6.9–7.3 and the ash content was 1.23–1.94%, which meet both the food standards where the permitted ash content is up to 2.0% (Food Chemical Codex 10) and pharmaceutical standards where the permitted ash content is up to 3.0% (United States Pharmacopoeia 35 NF 30; European Pharmacopoeia; Japanese Pharmacopoeia 15). Gelatines have a higher viscosity (6.8–7.3 mPa·s) and are suitable, for example, in the food industry for the production of deposited marshmallows, or as a binder and gelling agent for meat industry products or for coatings.

The observed technological conditions in the preparation of gelatines have an analogous effect on the yield of gelatines and their quality, as is the case with the production of gelatines from traditional raw materials (beef and pork hides/skins and bones, and fish) by established technologies, i.e., by processing the raw material in an acidic or alkaline environment [1]. By properly selecting the amount and time of action of the enzyme in the processing of the raw material and the appropriate extraction time, gelatines with the desired gel strength and viscosity can be prepared. In industrial practice, it will be advantageous to utilize the multiple fractionation of the raw material at increasing extraction temperatures (typically 60–100 °C), which will result in the preparation of different quality gelatines with optimum utilization of the starting material (maximal gelatine yield). The undissolved residue after the gelatine extraction can be processed by hydrolysis with a suitable proteolytic enzyme into a collagen hydrolysate, which can be used in the food industry (e.g., as nutritional supplements, thickeners, etc.) or as an additive (humectant) in the cosmetic industry.

From the study of the influence of process factors and from the optimization part of the biotechnological preparation of gelatines from chicken feet, it can be stated that gelatines with a gel strength of 220–320 Bloom, viscosity of 3.5–7.3 mPa·s, and an ash content of up to 2.0% can be prepared by a suitable combination of process factors (amount of enzyme, time of raw material enzyme treatment, time and temperature of gelatine extraction). These are gelatines belonging to the category of high-quality food and pharmaceutical gelatines (gel strength of 220–300 Bloom, viscosity of 2.0–7.5 mPa·s) complying with the strict standards prescribed in the European Union, US or Japan (Directorate General for Health and Consumer Protection of the European Commission, USDA, FDA or MHLW).

Chicken gelatines with a high gel strength and a viscosity of about 4.0–5.5 mPa·s are suitable, for example, for the production of gelatine desserts, confectionery products (e.g., gummy bears, extruded marshmallows), and are also perfect for use in the meat industry (aspics, binder for meat emulsions, ham, jellies), in the manufacture of dairy products (low-fat butter spreads, panna cotta, aerated desserts, yogurt-based products), or on frozen semi-finished or finished products. Conversely, gelatines with a lower viscosity (<4.0 mPa·s) are suitable for making chewing gums or sugar sanded/oiled jelly items. For the preparation of hard gelatine capsules, all the prepared chicken gelatines with a viscosity of 4.5–6.5 mPa·s are suitable; for producing soft gelatine capsules, gelatines with a viscosity of 2.5–4.5 mPa·s are sufficient. Due to the high gel strength, chicken gelatines may find various applications in the biomedical field, similar to gelatines produced from pork and bovine feedstock. For example, they can be used as hydrogel carriers for delivering bioactive macromolecules, producing membranes, microspheres, and nanoparticles, encapsulating carriers for the controlled release of biologically active substances, or delivering cell transplants for tissue repairs [37,38,39]. A high Bloom value has a positive effect on the mechanical properties of gelatine films [40]. In addition, Lai suggested that the gelatine Bloom value has an influence on cellular responses to gelatine material as well [41]. The Bloom value of chicken gelatines is a critical parameter for preparing mixtures with other biopolymers, above all with polysaccharides, to adjust the gelling/thickening properties of such mixtures for food and pharmaceutical applications [42].

## 4. Conclusions

Most commercial gelatines are made from beef, pork, or fish tissues; industrial processing is based on the acidic or alkaline processing of the feedstock. The presented technology focuses on the preparation of gelatines from by-product collagen raw materials derived from the slaughter of chicken (chicken feet). The innovative technology element brings the biotechnological processing of (purified) feedstock by commercial food endoprotease, which, in contrast to acidic (type A gelatines) or alkaline (type B gelatines) processing, has a variety of economic, technological, and environmental advantages. The feedstock is treated with a small amount of enzyme (0.1–0.8%, based on the dry raw material weight) in neutral environment at room temperature; and the processing time, e.g., compared to alkaline beef processing, is significantly reduced (from weeks to months to tens of hours). After enzymatic treatment and washing of the feedstock in water, a standard hot-water extraction of gelatine follows. By selecting the technological conditions, gelatines with high gel strength (220–320 Bloom) with an ash content of less than 2.0% can be prepared to meet food and pharmaceutical standards. The gelatine yield is 18–38% for the one-step extraction process, which are very good values; a more optimal utilization of the feedstock can then be achieved in practice by a multi-step extraction process. The observed process parameters in the preparation of chicken gelatines also affects the gelatine viscosity (3.5–7.3 mPa·s). The strength of gelatine gels (a key parameter for the application of gelatines) is not significantly altered by the change in process parameters, as all of the gelatines prepared belong to the gelatine category with a high gel strength (>220 Bloom). Chicken gelatines can be an alternative to beef, pork, and fish gelatines, as they meet halal and kosher requirements. For their excellent properties, they are suitable in many food applications; for example, for confectioneries, sweet desserts, dairy products, or meat products. They can also be used in the pharmaceutical field for manufacturing hard gelatine capsules (HGC) and soft gelatine capsules (SGC), as well as various applications in the biomedical field (hydrogels, membranes, carriers, and films).

## 5. Patents

From the work reported in this manuscript, the following patent resulted: Patent CZ 307665 – biotechnology-based production of food gelatine from poultry by-products.

## Figures and Tables

**Table 1:** The experimental design and the results of processing of chicken feet into gelatines (study of the influence of process factors).

Exp. No.	Factors under Study			Collagen Hydrolysate		Gelatine					Summary of the Process	
	A	B	C									
	Enzyme Addition (%)	Enzyme Treatment (h)	Extraction Time (h)	HY (%)	Ash_H_^a^ ± SD (%)	GY (%)	Ash_G_^a^ ± SD (%)	F ± SD (Bloom)	η ± SD (mPa·s)	pH ± SD	Y_∑_ (%)	MBE (%)
1	0.2	24	1	9.8	20.6 ± 0.1	20.1	1.35 ± 0.02	295 ± 3	6.9 ± 0.1	6.2 ± 0.1	29.9	2.0
2	0.2	24	4	10.0	21.8 ± 0.2	27.4	0.61 ± 0.03	273 ± 3	6.5 ± 0.1	6.4 ± 0.1	37.4	4.9
3	0.2	120	1	10.7	23.9 ± 0.1	24.1	1.66 ± 0.01	266 ± 2	5.9 ± 0.2	6.4 ± 0.1	34.8	5.9
4	0.2	120	4	11.0	24.7 ± 0.3	33.5	0.88 ± 0.03	263 ± 2	5.2 ± 0.1	6.0 ± 0.2	44.5	3.8
5	0.8	24	1	11.2	27.2 ± 0.1	36.5	0.93 ± 0.02	241 ± 2	5.1 ± 0.2	6.4 ± 0.1	47.7	2.5
6	0.8	24	4	11.1	28.3 ± 0.2	37.9	0.77 ± 0.02	235 ± 3	4.7 ± 0.1	6.4 ± 0.1	49.0	2.6
7	0.8	120	1	11.4	28.6 ± 0.1	38.3	1.61 ± 0.01	228 ± 2	3.7 ± 0.1	6.3 ± 0.2	49.7	1.6
8	0.8	120	4	11.5	29.4 ± 0.1	39.1	1.32 ± 0.01	206 ± 2	3.1 ± 0.1	6.1 ± 0.1	50.6	0.7
9	0.5	72	2.5	10.9	25.7 ± 0.2	35.4	1.53 ± 0.02	249 ± 3	6.5 ± 0.3	6.1 ± 0.1	46.2	2.6

^a^—based on dry matter; HY—hydrolysate yield; GY—gelatine yield; Y_∑_—total yield; Ash_H_—ash content in hydrolysate; Ash_G_—ash content in gelatine; F—gelatine gel strength; η—gelatine viscosity; MBE—mass balance error

**Table 2:** Analysis of variance of the experimental design for gelatine yield, gelatine gel strength, and gelatine viscosity (study of the influence of process factors).

	Degree of Freedom	Sum of Squares	Mean Squares	F-Value	P-Value
Response: Gelatine yield, GY (%) = 16.35 + 19.46 A + 0.0341 B + 1.575 C; R^2^ = 88.65					
Regression	3	338.71	112.905	13.01	0.008
Factor A (Enzyme addition)	1	272.61	272.611	31.42	0.002
Factor B (Enzyme treatment time)	1	21.45	21.451	2.47	0.177
Factor C (Gelatine extraction time)	1	44.65	44.651	5.15	0.073
Error	5	43.38	8.676		
Total	8	382.10			
Response: Gelatine gel strength, F (Bloom) = 315.85 – 77.92 A – 0.2109 B – 4.42 C; R^2^ = 97.20					
Regression	3	5542.4	1847.46	57.87	0.000
Factor A (Enzyme addition)	1	4371.1	4371.13	136.92	0.000
Factor B (Enzyme treatment time)	1	820.1	820.12	25.69	0.004
Factor C (Gelatine extraction time)	1	351.1	351.13	11.00	0.021
Error	5	159.6	31.93		
Total	8	5702.0			
Response: Gelatine viscosity, η (mPa·s) = 8.37–3.29 A—0.0138 B—0.175 C; R^2^ = 87.18					
Regression	3	11.87	3.955	11.33	0.011
Factor A (Enzyme addition)	1	7.80	7.801	22.35	0.005
Factor B (Enzyme treatment time)	1	3.51	3.511	10.16	0.025
Factor C (Gelatine extraction time)	1	0.55	0.551	1.58	0.264
Error	5	1.75	0.349		
Total	8	13.61			

**Table 3:** The experimental design and the results of processing of chicken feet into gelatines (process optimization).

Exp. No.	Factors under Study		Gelatine				
	A	C					
	Enzyme Addition (%)	Extraction Time (min)	GY (%)	Ash_G_^a^ ± SD (%)	F ± SD (Bloom)	η ± SD (mPa·s)	pH ± SD
10	0.1	15	17.0	1.31	325	7.3	7.3
11	0.1	45	18.9	1.23	315	7.2	7.2
12	0.4	15	20.6	1.94	308	6.9	7.2
13	0.4	45	21.2	1.58	301	6.8	6.9
14	0.25	30	19.8	1.45	310	6.9	7.2

^a^—based on dry matter; GY—gelatine yield; Ash_G_—ash content in gelatine; F—gelatine gel strength; η—gelatine viscosity
